# The Columbia Behavior Scale for Dementia: Validity and Reliability in Long-Term Care Settings

**DOI:** 10.1093/geroni/igaa057.598

**Published:** 2020-12-16

**Authors:** William Mansbach, Ryan Mace

**Affiliations:** Mansbach Health Tools, LLC, Simpsonville, Maryland, United States

## Abstract

Numerous neuropsychiatric inventories have been created for behavioral and psychological symptoms of dementia (BPSD). These inventories are seldom used in long-term care (LTC) settings due to questionable psychometrics, lengthy administration, and reliance on knowledgeable informants. The Columbia Behavior Scale for Dementia (CBS) is a rapidly administered BPSD rating tool that was developed for LTC residents. The 11-item CBS can be completed in less <5 minutes independently, with nursing staff, or in conjunction with interdisciplinary care teams. LTC residents (N = 350) participated in a validation study in Maryland, USA (M age = 78.38, SD = 10.82). Internal consistency (⍺ = 0.75) and inter-rater reliability (r = 0.99) for the CBS were strong. CBS scores were not biased by informant type (p > 0.05): GNAs/CNSs (40.69%), nurses (36.10%), other facility staff (23.21%). Diagnostic validity was confirmed by significantly higher CBS scores (p < 0.001; large effect, d = .63) for LTC residents with dementia (n = 197, M = 4.63, SD = 4.58) versus those without dementia (n = 145, M = 2.17, SD = 2.87). Higher CBS scores were significantly associated with greater impairment on cognitive instruments (r range = -0.25, -.36) and increased mood dysfunction (r range = 0.20, 0.26), indicating convergent validity. Principal components analysis produced three CBS factors, psychosis, aggression, and non-aggressive motor disinhibition, which significantly identified LTC residents with greater odds for antipsychotic use. Results will be discussed in terms of right-sizing antipsychotic utilization, improving nonpharmacological behavior management, and enhancing the dementia literacy of nursing staff.

